# Knowledge and Practice of Anemia Prevention Strategies in Pregnancy Among Women Attending Antenatal Care in Northeast Ethiopia, 2024: A Cross‐Sectional Study

**DOI:** 10.1155/anem/4969932

**Published:** 2026-08-30

**Authors:** Zenebe Tefera Ayele, Mandefro Assefaw Geremew, Wondimnew Gashaw Kettema, Nigusie Abebaw Mekonen, Atrsaw Dessie Liyew, Tenagnework Dilnesa Mulualem, Lemlem Chanyalew Melese, Asres Eshetie Feleke, Lubaba Ahmed Mohamed, Yezbalem Negesse Simegn, Alemtsehay Wossen Samuel, Mengistu Abate Belay

**Affiliations:** ^1^ Department of Midwifery, College of Medicine and Health Sciences, Wollo University, Dessie, Ethiopia, wu.edu.et; ^2^ Department of Epidemiology and Biostatistics, College of Medicine and Health Sciences, Wollo University, Dessie, Ethiopia, wu.edu.et

**Keywords:** anemia prevention, antenatal care, Ethiopia, knowledge, practice, pregnant women, South Wollo

## Abstract

**Background:**

Anemia during pregnancy is considered one of the main risk factors contributing to 20%–40% of maternal mortality. Most causes of anemia in pregnancy are preventable. However, data on women’s knowledge and practices regarding comprehensive strategies necessary for anemia prevention during pregnancy are limited in Ethiopia, particularly in the study area. Therefore, this study aimed to assess the knowledge and practice of anemia prevention strategies during pregnancy in South Wollo Zone, Northeastern Ethiopia.

**Methods:**

A hospital‐based cross‐sectional study design was employed from November 1 to December 10, 2024, among 420 pregnant women. A systematic sampling technique was used to select the study participants. Data were analyzed using SPSS Version 25. Descriptive statistics and generalized estimating equations with a binary logistic model were performed to assess the net effects of predictors while controlling for confounders and computing cluster‐robust standard errors. Statistical differences were considered at *p*‐value < 0.05, and the strength of association was assessed by odds ratio and respective cluster‐robust 95% confidence intervals.

**Result:**

The study showed that 52.1% of pregnant women had good knowledge of anemia prevention strategies and 53.1% demonstrated good practice. Good knowledge was significantly associated with higher maternal education (AOR = 2.04, 95% CI: 1.11, 3.76), higher partner education (AOR = 3.19, 95% CI: 2.24, 4.57), and intended pregnancy (AOR = 3.78, 95% CI: 1. 41, 10.21). Good practice was significantly associated with urban residence (AOR = 2.98, 95% CI: 1.59, 5.63), higher maternal education (AOR = 2.17, 95% CI: 1. 19, 3.96), previous history of anemia (AOR = 3.20, 95% CI: 1.33, 7.72), and good knowledge (AOR = 2.01, 95% CI: 1. 32, 3.06).

**Conclusion:**

Just over half of the pregnant women in South Wollo Zone had good knowledge and practice regarding anemia prevention, leaving substantial, critical gaps in dietary habits, deworming, and bed net utilization. Therefore, healthcare providers and program managers should focus on reaching rural and less‐educated women with targeted antenatal education, while expanding preconception family planning services to reduce unintended pregnancies.

## 1. Introduction

Anemia is a public health concern affecting 36.5% of pregnant women globally, with the highest burden in low‐ and middle‐income countries, including Ethiopia [1, 2]. Anemia during pregnancy contributes to 20%–40% of maternal deaths, both directly and indirectly [3]. In Ethiopia, the national prevalence of anemia in pregnancy is 31.66% [4]. While the consequences of anemia, such as miscarriage, stillbirth, preterm birth, low birth weight, low Apgar scores, infant death, increased incidence of cesarean section, and elevated maternal mortality are well documented [1, 5–9], women’s knowledge (their understanding of which behaviors prevent anemia) and their practice (translation of their understanding into daily action) are less frequently studied.

Globally, adequate knowledge and preventive practices regarding anemia remain suboptimal, with widely varied reports across low‐ and middle‐income countries. In Nepal, only 48.7% of women demonstrated good knowledge of anemia prevention strategies, and lower proportion (34.0%) of women engaged in appropriate preventive practices [10]. In Nigeria, the proportion of women with good knowledge ranges from 56.5% to 81.2%, while those with good practice ranges from 50.0% to 66.9% [11–13]. In Ethiopia, good knowledge and practice levels are generally within 48%–57% and 46%–50%, respectively [14–16]. These evidences reveal a persistent knowledge and practice gap, where a substantial proportion of women either lack essential preventive knowledge or, despite having knowledge, fail to translate it into consistent preventive actions. Knowledge and practice gaps in anemia prevention strategies have both clinical and programmatic consequences, such as perpetuating anemia across pregnancies and adverse maternal outcomes, increase healthcare costs, and undermine Ethiopia’s national targets, such as reducing anemia among women by 58% (from 24% of prevalence to 10%) by 2030 [16, 17].

Although few facility‐based studies have been conducted in Ethiopia, no published study has systematically assessed both knowledge and practice of the complete range of World Health Organization’s recommended anemia prevention strategies, including iron folate, diet, deworming, ITN use, avoidance of dietary inhibitors, and family planning among pregnant women [18], particularly in Northeast Ethiopia. Thus, this study aimed to assess knowledge and practice of anemia prevention strategies among pregnant women attending antenatal care in northeast Ethiopia.

## 2. Materials and Methods

### 2.1. Study Design, Setting, and Period

An institution‐based cross‐sectional study design was employed among pregnant women attending antenatal care at selected public Hospitals in South Wollo Zone, Northeastern Ethiopia from November 1 to December 10, 2024. The South Wollo Zone is one of the 12 zones in the Amhara Region of Ethiopia. There are 14 public hospitals in the South Wollo Zone. These hospitals are serving 2,243,013 populations.

### 2.2. Source and Study Population

The source population consisted of all pregnant women attending antenatal care services at public hospitals in the South Wollo Zone. The study population comprised all pregnant women who attended antenatal care clinics at the five selected public hospitals during the data collection period and met the inclusion criteria.

### 2.3. Inclusion and Exclusion Criteria

All pregnant women who came for antenatal care visit in the selected hospitals during the study period were included, and those with known mental illness (unable to provide informed consent) were excluded from the study.

### 2.4. Sample Size and Sampling Procedure

**FIGURE 1:**
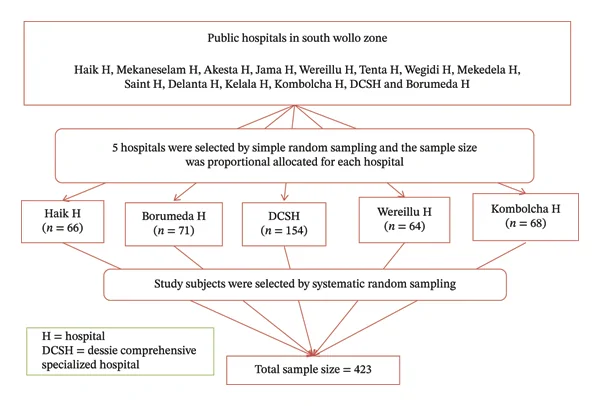
Schematic presentation of the sampling procedure for the study on knowledge and practice of anemia prevention strategies in pregnancy among women attending antenatal care in northeast Ethiopia, 2024.

The required sample size (*n*) was determined using a single population proportion formula considering the following assumptions: anemia prevention practice (*p* = 50%) from the study conducted in Western Ethiopia [15]; confidence level of (*Z*) = 95%; 5% of the margin of error (*d*); $n=Z_{\alpha /2}^{2}p\left(1-p\right)/d^{2}\;n={\left(1.96\right)}^{2}0.50.5384\times /{\left(0.05\right)}^{2}=$ and 10% nonresponse rate, the final sample size was 423. Of the 14 public hospitals in South Wollo, five were selected using a simple random sampling technique: Haik Primary Hospital, Dessie Comprehensive Specialized Hospital, Boru Meda General Hospital, Wereillu Primary Hospital, and Kombolcha General Hospital. The number of study participants for hospital was allocated proportionally based on each hospital’s average monthly antenatal care case flow, calculated from the three preceding consecutive months. Based on this, the total average monthly case flow of the five selected Hospitals was 678. A systematic random sampling technique was then used to select individual study participants. First, the sampling interval (*k*) was determined using formula *K* = *N*/*n*, where N is the total estimated monthly case flow (678) and *n* is the total sample size (423); therefore, *k* = 678/423 = 1.6∼2. The first pregnant woman was selected using a simple lottery method from the first two women who arrived on the first day of data collection at each hospital. Thereafter, every second pregnant woman attending antenatal care was selected until the required sample size for each hospital was achieved (Figure 1).

### 2.5. Variables of the Study

#### 2.5.1. Dependent Variables

• Knowledge of anemia prevention
• Practice for anemia prevention

#### 2.5.2. Independent Variables

• Sociodemographic variable: Age, residence, marital status, religion, educational status, and occupational status, husband’s educational status, husband’s occupational status
• Obstetric‐related variables: Gravidity, parity, number of ANC visit, gestational age, pregnancy intention, ANC attendant, history of anemia

### 2.6. Operational Definition

• Knowledge of anemia prevention strategies was measured using 10‐item questionnaires. The calculated mean score was 3.91, and women who scored at or above the mean score were classified as having “good knowledge,” while those who scored below the mean were classified as having “poor knowledge.”
• Practice of anemia prevention strategies was measured using a 7‐item Likert scale, with a maximum obtainable score of 35. The mean practice score of the participants was 21.97 (out of a maximum of 35). Respondents who scored ≥ 21.97 were categorized as having good practice, while those who scored < 21.97 were categorized as having poor practice.

### 2.7. Data Collection Procedure

The data were collected through face‐to‐face interviews using a pretested, structured questionnaire. The questionnaire was adapted from validated instruments in existing literature, and it includes information related to the sociodemographic characteristics, obstetric history, question used to assess knowledge, and practice of anemia prevention strategies [10, 12, 13, 16, 19]. The internal consistency of the measurement scales was evaluated using the final collected dataset. The instrument demonstrated good reliability, with a Cronbach’s alpha of 0.83 for the 10‐item knowledge scale and acceptable reliability with a Cronbach’s alpha of 0.789 for the 7‐item practice scale. The data were collected by five trained nurses using the Amharic version of the questionnaire.

### 2.8. Data Quality Control

The questionnaire was prepared originally in English and was translated to Amharic (local language) and back to English by two independent persons to keep the consistency of the questionnaire. Training was given to data collectors and supervisors. A pretest was conducted on 5% (12 pregnant mothers), and necessary modifications were made before the actual data collection.

### 2.9. Data Processing and Analysis

The collected data were checked for completeness and consistency before analysis and were entered into Epi Data version 4.6. The data were exported to SPSS version 25 software for analysis. Descriptive statistics were used to categorize and summarize the data. Bivariable logistic regression analysis was done to assess the crude association between each independent and outcome variable. Variables with a *p*‐value < 0.25 in bivariable analysis were entered into a multivariable modeling. Generalized estimating equations (GEE) with a binary logistic model were used to assess the net effects of the predictors by controlling for confounders while computing cluster‐robust standard errors. Variables with a *p* < 0.05 in the final GEE models were considered as statistically significant factors for the outcome variables. The adjusted odds ratio (AOR) with cluster‐robust 95% confidence interval (CI) was used to measure the strength of association. Model fitness was checked using the quasi‐likelihood under the independence model criterion (QIC). The knowledge model demonstrated a stable fit with a QIC of 561.28, and the practice model showed an equally robust fit with a QIC of 562.91. Multicollinearity among the independent variables was assessed using the variance inflation factor (VIF), which revealed no significant multicollinearity in either analysis. For the knowledge model, VIF values ranged from 1.017 to 1.886 (mean VIF = 1.241), while the VIF values for the practice model range from 1.019 to 1.299 (mean VIF = 1.103).

## 3. Results

### 3.1. Sociodemographic Characteristics of the Respondents

A total of 420 pregnant women participated in the study, yielding a response rate of 99.3%. The largest proportion of participants (62.4%) were aged 25–34. Over half of the women (57.9%) resided in urban areas. Religiously, 57.1% identified as Muslim, and an overwhelming majority (99.3%) were married. In terms of educational attainment, 32.4% had completed primary education, while nearly a quarter (22.6%) had no formal education. The occupational distribution included 31.0% farmers, followed by housewives (22.4%). Regarding spousal characteristics, 41.7% of the participants’ husbands had a secondary education, and 31.0% were farmers (Table 1)

**TABLE 1:** Sociodemographic characteristics of respondents in South Wollo, Ethiopia, 2024.

Variable	Response	Frequency	Percentage
Age	15–24	90	21.4
	25–34	262	62.4
	35–49	68	16.2

Residence	Urban	243	57.9
	Rural	177	42.1

Religion	Orthodox	180	42.9
	Muslim	240	57.1

Marital status	Single	2	0.5
	Married	417	99.3
	Divorced	1	0.2

Educational status	No formal education	95	22.6
	Primary school	136	32.4
	Secondary school	101	24.0
	College and above	88	21.0

Occupation status	Housewife	94	22.4
	Government employee	83	19.8
	Merchant	86	20.5
	Farmer	130	31.0
	Other^a^	27	6.4

Husband’s educational status	No formal education	49	11.7
	Primary school	97	23.1
	Secondary school	175	41.7
	College and above	99	23.6

Husband’s occupational status	Government employee	90	21.4
	Private employee	59	14.0
	Merchant	61	14.5
	Farmer	130	31.0
	Other^b^	80	19.0

^a^Student, daily labor.

^b^Daily labor, driver, and carpenter.

### 3.2. Obstetric‐Related Characteristics

The majority of women were multigravida (70.0%). Most pregnancies were reported as planned (93.3%). In terms of gestational age, 42.9% were in the second trimester and 47.6% in the third trimester. A significant proportion of women (68.3%) had attended their third or more ANC visit. Midwives were the primary ANC providers for the vast majority (83.6%). Only a small fraction of the women (10.7%) reported a previous history of anemia (Table 2).

**TABLE 2:** Obstetric‐related characteristics of respondents in South Wollo, Ethiopia, 2024.

Variable	Responses	Frequency	Percentage
Gravidity	Primigravida	126	30.0
	Multigravida	294	70.0

Parity	Nullipara	40	9.5
	Primipara	101	24.0
	Multipara	153	36.4

Pregnancy intention	Intended	392	93.3
	Unintended	28	6.7

Gestational age by trimesters	1st trimester	40	9.5
	2nd trimester	180	42.9
	3rd trimester	200	47.6

Number of ANC visit	1st time	44	10.5
	2nd time	89	21.2
	≥ 3rd time	287	68.3

ANC attendant	Midwife	351	83.6
	Nurse	6	1.4
	IESO	14	3.3
	Resident students	49	11.7

Previous history of anemia	Yes	45	10.7
	No	375	89.3

### 3.3. Knowledge of Anemia Prevention Strategies During Pregnancy

As shown in the knowledge assessment, 52.1% (95% CI: 47.2%, 57.0%) of the pregnant women demonstrated good overall knowledge regarding anemia prevention strategies. While general awareness was high, with 81.0% having heard of anemia in pregnancy, knowledge on specific preventive measures was variable. Most women (75.5%) correctly identified iron supplementation as a preventive measure. However, critical knowledge gaps were evident: only 15.5% knew about avoiding tea/coffee/milk around meals, 21.9% were aware of the role of deworming, and 43.3% recognized the use of insecticide‐treated bed nets as an anemia prevention strategy (Table 3)

**TABLE 3:** Knowledge of anemia prevention strategies during pregnancy in South Wollo, Ethiopia, 2024.

Variable	Yes (%)	No (%)
Heard of anemia in pregnancy	340 (81.0)	80 (19.0)
Know anemia is decreased RBC/hemoglobin	34 (8.1)	386 (91.9)
Aware iron supplements prevent anemia	317 (75.5)	103 (24.5)
Know avoid coffee/tea/milk near meals	65 (15.5)	355 (84.5)
Aware high‐fiber meals (barley, lentils) prevent anemia	75 (17.9)	345 (82.1)
Know red meat, liver, greens prevent anemia	98 (23.3)	322 (76.7)
Aware family planning impacts anemia	240 (57.1)	180 (42.9)
Know deworming in pregnancy prevents anemia	92 (21.9)	328 (78.1)
Aware insecticide‐treated nets prevent anemia	182 (43.3)	238 (56.7)
Know antenatal care prevents anemia	258 (61.4)	162 (38.6)

### 3.4. Women’s Practices Regarding Anemia Prevention During Pregnancy

The study found that 53.1% (95% CI: 48.3%, 57.8%) of the pregnant women had good practice scores for anemia prevention. The practice of iron‐folate supplementation was commendable, with 89.1% reporting taking supplements “often” or “always.” Dietary practices were moderate, with 58.3% “often” eating three or more balanced meals daily. Less than one‐third (29.8%) of participants reported using an insecticide‐treated bed net, and the majority (92.9%) of them had never received deworming treatment during their current pregnancy (Table 4)

**TABLE 4:** Women’s practices regarding anemia prevention during pregnancy, in South Wollo, Ethiopia, 2024.

Variables	Never *N* (%)	Rarely *N* (%)	Sometimes *N* (%)	Often *N* (%)	Always *N* (%)
Consistently taking iron‐folate supplements	3 (0.7)	0 (0.0)	43 (10.2)	188 (44.8)	186 (44.3)
Consuming iron‐rich foods	0 (0.0)	85 (20.2)	196 (46.7)	116 (27.6)	23 (5.5)
Eating 3+ balanced meals with fiber	0 (0.0)	0 (0.0)	91 (21.7)	245 (58.3)	84 (20.0)
Avoiding tea/coffee/milk during meals	102 (24.3)	111 (26.4)	78 (18.6)	82 (19.5)	47 (11.2)
Using insecticide‐treated bed nets	295 (70.2)	85 (20.2)	18 (4.3)	22 (5.2)	0 (0.0)
Receiving deworming treatment	390 (92.9)	7 (1.7)	0 (0.0)	10 (2.4)	13 (3.1)
Attending regular antenatal care	1 (0.2)	0 (0.0)	80 (19.0)	122 (29.0)	217 (51.7)

### 3.5. Factors Associated With Knowledge of Anemia Prevention Among Pregnant Women

In the GEE multivariable analysis, educational status, partner’s educational status, and pregnancy intention were significantly associated with good knowledge of anemia prevention in pregnancy. Pregnant women with a college and above education were 2.04 times (AOR = 2.04, 95% CI: 1.11, 3.76) more likely to have good knowledge of anemia prevention strategies compared to those with no formal education. Pregnant women whose partner’s educational status was college and above were 3.19 times (AOR = 3.19, 95% CI: 2.24, 4.57) more likely to have good knowledge of anemia prevention strategies compared to their counterparts. Similarly, women with a intended pregnancy were 3.78 times (AOR = 3.78, 95% CI: 1. 41, 10.21) more likely to have good knowledge than those with an unplanned pregnancy (Table 5)

**TABLE 5:** Factors associated with knowledge of anemia prevention among pregnant women, in South Wollo, Ethiopia, 2024.

Variables	Knowledge		COR (95% CI)	AOR (95% CI)
	Poor (%)	Good (%)		
Age				
15–24	44 (48.9)	46 (51.1)	1	1
25–34	135 (51.5)	127 (48.5)	0.90 (0.55, 1.45)	0.80 (0.63, 1.01)
35–49	22 (32.4)	46 (67.6)	2.00 (1.03, 3.85)	1.64 (0.81, 2.21)
Residence				
Urban	98 (40.3)	145 (59.7)	2.05 (1.38, 3.05)	1.39 (0.94, 2.06)
Rural	103 (58.2)	74 (41.8)	1	1
Educational status of the woman				
No formal education	58 (61.1)	37 (38.9)	1	1
Primary school	64 (47.1)	72 (52.9)	1.76 (1.03, 3.00)	1.52 (0.74, 3.13)
Secondary school	47 (46.5)	54 (53.5)	1.80 (1.02, 3.17)	1.23 (0.74, 2.06)
College and above	32 (36.4)	56 (63.6)	2.74 (1.51, 4.99)	2.04 (1.11, 3.76)^∗^
Partners education				
No formal education	34 (69.4)	15 (30.6)	1	1
Primary school	53 (54.6)	44 (45.4)	1.88 (0.91, 3.89)	1.10 (0.64, 2.69)
Secondary school	81 (46.3)	94 (53.7)	2.63 (1.33, 5.17)	1.11 (0.89, 3.95)
College and above	33 (33.3)	66 (66.7)	4.53 (2.16, 9.47)	3.19 (2.24, 4.57)^∗^
Gravidity				
Primigravida	60 (47.6)	66 (52.4)	1	1
Multigravida	141 (48.0)	153 (52.0)	0.98 (0.65, 1.49)	1.08 (0.74, 1.57)
Pregnancy intention				
Intended	180 (45.9)	212 (54.1)	3.53 (1.46, 8.50)	3.78 (1.41, 10.21)^∗^
Unintended	21 (75.0)	7 (25.0)	1	1
Number of ANC visit				
1st time	26 (59.1)	18 (40.9)	1	1
2nd time	43 (48.3)	46 (51.7)	1.54 (0.74, 3.21)	1.87 (0.85, 4.15)
≥ 3rd time	132 (46.0)	155 (54.0)	1.69 (0.89, 3.23)	2.09 (0.93, 4.71)
Previous history of anemia				
Yes	16 (35.6)	29 (64.4)	1.76 (0.92, 3.35)	1.70 (0.85, 2.69)
No	185 (49.3)	190 (50.7)	1	1

*Note:* 1 = Reference.

Abbreviations: AOR, adjusted odds ratio; CL, confidence interval; COR, crude odds ratio.

^∗^ *p* value < 0.05.

### 3.6. Factors Associated With Anemia Prevention Practices Among Pregnant Women

The GEE multivariable analysis revealed that place of residence, educational status, previous history of anemia, and level of knowledge were significant predictors of good practice on anemia prevention strategies. Urban residents were 2.98 times (AOR = 2.98, 95% CI: 1.59, 5.63) more likely to have good practice compared to rural residents. Women with college and above educational level had 2.17 times (AOR = 2.17, 95% CI: 1. 19, 3.96) higher odds of good practice than those with no formal education. Those with a previous history of anemia were 3.20 times (AOR = 3.20, 95% CI: 1.33, 7.72) more likely to practice preventive measures. Crucially, women with good knowledge were 2.01 times (AOR = 2.01, 95% CI: 1. 32, 3.06) more likely to have good practice compared to their counterparts (Table 6).

**TABLE 6:** Factors associated with anemia prevention practices among pregnant women in South Wollo, Ethiopia, 2024.

Variables	Practice		COR (95% CI)	AOR (95% CI)
	Poor (%)	Good (%)		
Age				
15–24	48 (53.3)	42 (46.7)	1	1
25–34	119 (45.4)	143 (54.6)	1.37 (0.85, 2.22)	1.51 (0.57, 3.98)
35–49	30 (44.1)	38 (55.9)	1.44 (0.76, 2.72)	0.81 (0.36, 1.85)
Residence				
Urban	89 (36.6)	154 (63.4)	2.71 (1.81, 4.03)	2.98 (1.59, 5.63)^∗∗^
Rural	108 (61.0)	69 (39.0)	1	1
Educational status				
No formal education	55 (57.9)	40 (42.1)	1	1
Primary school	71 (52.2)	65 (47.8)	1.25 (0.74, 2.13)	0.88 (0.64, 1.21)
Secondary school	48 (47.5)	53 (52.5)	1.51 (0.86, 2.66)	0.66 (0.46, 1.97)
College and above	23 (26.1)	65 (73.9)	3.88 (2.07, 7.26)	2.17 (1.19, 3.96)^∗^
Gravidity				
Primigravida	63 (50.0)	63 (50.0)	1	1
Multigravida	134 (45.6)	160 (54.4)	1.19 (0.78, 1.81)	1.57 (0.90, 2.26)
Number of ANC visit				
1st time	25 (56.8)	19 (43.2)	1	1
2nd time	43 (48.3)	46 (51.7)	1.40 (0.68, 2.91)	1.56 (0.52, 4.66)
≥ 3rd time	129 (44.9)	158 (55.1)	1.61 (0.85, 3.05)	2.07 (0.84, 5.14)
Previous history of anemia				
Yes	11 (24.4)	34 (75.6)	3.04 (1.49, 6.18)	3.20 (1.33, 7.72)^∗^
No	186 (49.6)	189 (50.4)	1	1
Knowledge				
Poor	117 (58.2)	84 (41.8)	1	1
Good	80 (36.5)	139 (63.5)	2.42 (1.63, 3.58)	2.01 (1.32, 3.06)^∗∗^

*Note:* ANC = antenatal care, 1 = reference.

Abbreviations: AOR, adjusted odds ratio; CI, confidence interval; COR, crude odds ratio.

^∗^ *p* value < 0.05.

^∗∗^ *p* value < 0.001.

## 4. Discussion

This study assessed the comprehensive knowledge and practice of anemia prevention strategies among pregnant women in South Wollo Zone, Northeast Ethiopia. The finding demonstrated that 52.1% (95% CI: 47.2%, 57.0%) of pregnant women possessed a good knowledge base, while 53.1% (95% CI: 48.3%, 57.8%) of women reported good preventive practice. This finding highlights a critical gap, revealing that nearly half of the study participants either lack sufficient knowledge or have not fully adopted the multipronged anemia prevention strategies recommended by the WHO and the Ethiopian Ministry of Health.

The observed knowledge level(52.1%) was in line with previous studies conducted in Western Ethiopia (57.3%), Nigeria (56.5%), and Nepal (48.7%) [10, 13, 15]. However, the finding of this study was lower than previous studies conducted at a University Teaching Hospital in Nigeria (68.89%), in South‐West Nigeria (81.2%) [13, 20]. This disparity likely attributed to structural differences in how health systems deliver care. Many facilities in Nigeria use dedicated group health education blocks before individual checkups, which significantly boosts how much information mothers retain [20]. By contrast, public hospitals in Ethiopia rely on one‐on‐one counseling. This model easily breaks down under heavy patient loads, forcing midwives to treat clinical visits as rushed, task‐oriented pill distribution sessions [21, 22]. As a result, critical topics like deworming schedules and dietary interactions get sidelined, mirroring national data where giving out supplements consistently takes priority over actual nutritional counseling [23].

The current study reveals that multiple factors, such as women’s educational level, partner’s education, and pregnancy intention, emerged as key predictors of knowledge regarding the prevention of anemia. Accordingly, women with higher education (college and above) were 2.04 times more likely to demonstrate good knowledge of anemia prevention strategies. A similar trend was reported in Western Ethiopia, where women with higher educational attainment had 5.03 times higher odds of possessing good knowledge [15]. The observed association indicates that education enhances health literacy, critical thinking, and access to information through various channels [24].

Women whose partners had a college and above education had 3.19 times higher odds of good knowledge. This indicates that the influential role of husbands in household health decision‐making and information sharing in this cultural context is an often‐overlooked channel for health promotion. Furthermore, the study shows that women with intended pregnancies were 3.78 times more likely to have good knowledge of anemia prevention strategies. This finding aligns with behavioral theory suggesting that intended pregnancies are associated with greater pre‐conception care‐seeking, proactive information gathering, and overall health consciousness [25, 26].

The observed level of practice (53.1%) was in line with previous studies conducted in Western Ethiopia where 50% of pregnant women had good practice of anemia prevention [15]. On the other hand, the finding was lower than previous studies conducted at a University Teaching Hospital in Nigeria (66.9%) and Saudi Arabia (60%) [13, 27]. However, the finding of this study was higher than previous studies conducted in Nepal (34%) and Ghana (39.1%) [10, 28]. The observed differences in the level of practice toward anemia prevention strategies across studies may be primarily attributed to variations in healthcare infrastructure, cultural beliefs, and public health initiatives, which may influence access to education, resources, and support for anemia prevention, resulting in significant disparities in adherence rates among pregnant women.

The study reveals significant disparities in the practice of various strategies. The reported adherence to iron‐folate supplementation at 89.1% is commendable and aligns with findings from studies in Western Ethiopia [29], indicating successful program delivery. However, this contrasts sharply with the critically low rates of other preventive measures: 70.2% of women never used bed nets, and 92.9% never received deworming treatment. This disparity aligns with previous findings from Ethiopia and Nigeria, where nutritional practices often take precedence over other interventions [13, 29]. The justification for this gap may be multifaceted. Iron tablets are typically provided free during ANC, creating a structural enabler, whereas bed nets may require separate procurement and deworming may not be routinely offered or emphasized. The implication is that high adherence to one intervention does not guarantee comprehensive prevention.

The study also identified factors associated with women’s level of practice of anemia prevention during pregnancy. Accordingly, women who live in urban were 2.98 times more likely to demonstrate good anemia prevention practices. This strong association is consistent with a prior study conducted in Northwest Ethiopia, which reported that urban residents were 4.5 times more likely to demonstrate good anemia prevention practices [16]. The observed association may be attributed to the fact that women living in urban areas have greater exposure to health information through various media and a higher utilization of health services [30].

The study also shows that women with a college education or higher were 2.17 times more likely to demonstrate good anemia prevention practices. A similar trend was observed in Northwest Ethiopia and Western Ethiopia, where higher educational level was associated with 2.39 and 3.67 times in the likelihood of good prevention practice, respectively [15, 16]. A possible explanation for the observed association is that higher educational levels in mothers may enhance knowledge, health literacy, and access to resources, leading to better practices in anemia prevention.

Similarly, women who had a previous history of anemia were 3.2 times more likely to demonstrate good anemia prevention practice. This finding may be supported by the idea that personal experience with illness motivates behavioral change [31]. However, no significant association was observed between a history of anemia and women’s anemia prevention practice in Western Ethiopia [15].

We also observed that women with good knowledge of anemia prevention strategies had twice the odds of demonstrating good anemia prevention practices. This aligns with regional evidence from Northwest and Western Ethiopia, where knowledgeable mothers exhibited 2.54 and 4.93 times higher odds of anemia prevention practices [15, 16]. This implies that enhancing women’s knowledge could improve their adherence to effective practices, which suggests that educational interventions and targeted health communication efforts are crucial for reducing anemia prevalence in these populations, ultimately leading to better maternal and child health outcomes.

### 4.1. Strength and Limitations

This study focuses on simple iron supplementation to evaluate how mothers understand and stick to the complete WHO anemia prevention bundle. Sampling randomly across five public hospitals gives a strong representative, while GEE log‐linear models allowed us to account for clustering and confounding variables.

However, there are clear limitations. The cross‐sectional design of the study cannot establish direct cause‐and‐effect between factors and outcomes. We also relied on maternal self‐reports, which are naturally vulnerable to recall and social desirability biases that can inflate actual compliance. Finally, without objective clinical biomarkers like serial hemoglobin testing, we could not track the real‐time biological impact of these practices across trimesters.

## 5. Conclusion

In conclusion, the findings of this study revealed that just over half of the pregnant women in the South Wollo Zone possess good knowledge and preventive practices regarding anemia prevention during pregnancy. However, major gaps persist in dietary behaviors, bed net use, and deworming uptake. Higher educational attainment for both the woman and her partner, along with having an intended pregnancy were significant factors of good knowledge, whereas living in an urban area, having a higher formal education, a history of anemia, and maintaining good knowledge were strong independent predictors of good anemia prevention practices. Therefore, healthcare providers and program managers should focus on reaching rural and less‐educated women with targeted antenatal education, while expanding preconception family planning services to reduce unintended pregnancies, and implement focused educational campaigns that directly address the critical gaps in deworming, bed net utilization, and dietary practices habits.

## Author Contributions

Zenebe Tefera Ayele was involved in project designing, proposal development, project supervision, data collection and analysis, and write‐up and submission of the manuscript. All other authors equally involved in designing of the project, proposal development, project supervision, and write‐up of the manuscript.

## Funding

The data collection was funded by Wollo University.

## Disclosure

The funder had no role in the study design, data entry and analysis, decision to publish, or preparation of the manuscript.

## Ethics Statement

The study was ethically approved by Research and Ethical Review Board of Wollo University, College of Medicine and Health Sciences. Written informed consent was obtained from individual respondents.

## Conflicts of Interest

The authors declare no conflicts of interest.

## Data Availability

The data that support the findings of this study are available from the corresponding author upon reasonable request.
